# Exophthalmos: A Forgotten Clinical Sign of Cushing's Syndrome

**DOI:** 10.1155/2013/205208

**Published:** 2013-03-10

**Authors:** Aldo Schenone Giugni, Shylaja Mani, Subramanian Kannan, Betul Hatipoglu

**Affiliations:** ^1^Department of Internal Medicine, Cleveland Clinic Foundation, 9500 Euclid Avenue Desk NA10, Cleveland, OH 44195, USA; ^2^Department of Endocrinology, Diabetes and Metabolism, Cleveland Clinic Foundation, 9500 Euclid Avenue Desk F20, Cleveland, OH 44195, USA

## Abstract

Exophthalmos is typically associated with Graves' ophthalmopathy. Although originally described by Harvey Cushing, exophthalmos is an underappreciated sign of Cushing's syndrome. We present a case of a 38-year-old female who presented with severe bilateral proptosis and was subsequently diagnosed with Cushings disease. We discuss the possible mechanisms causing exophthalmos in patients with either endogenous or exogenous hypercortisolemia.

## 1. Case Presentation

**Figure 1:**
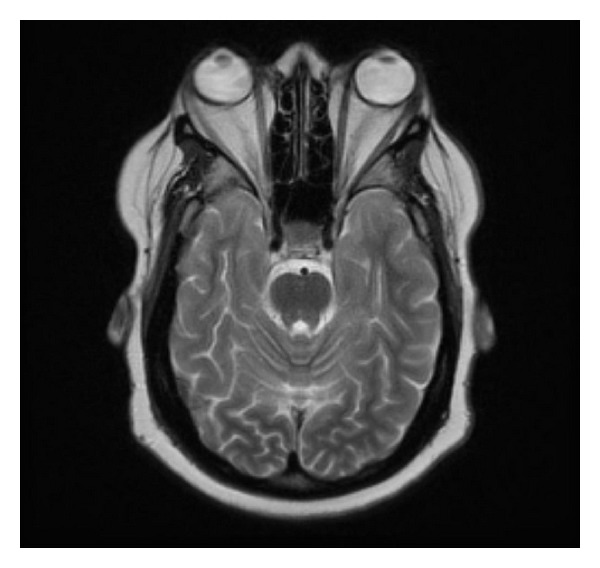
Bilateral exophthalmos as seen on MRI.

**Figure 2:**
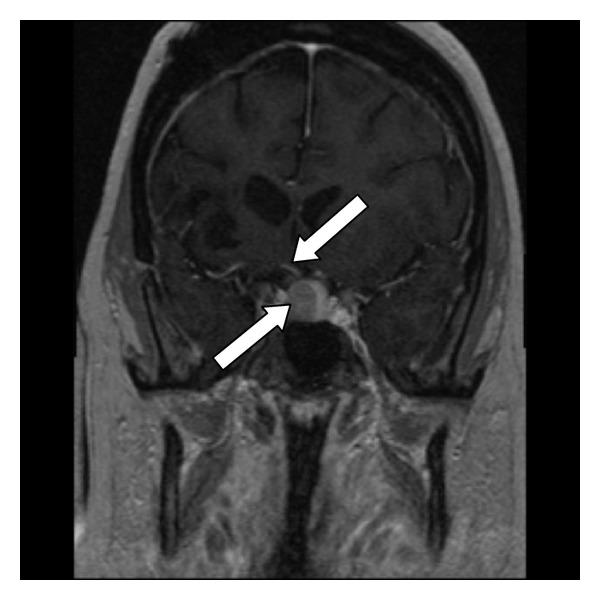
MRI of pituitary (coronal view) (arrows showing the macroadenoma and stalk deviation).

**Figure 3:**
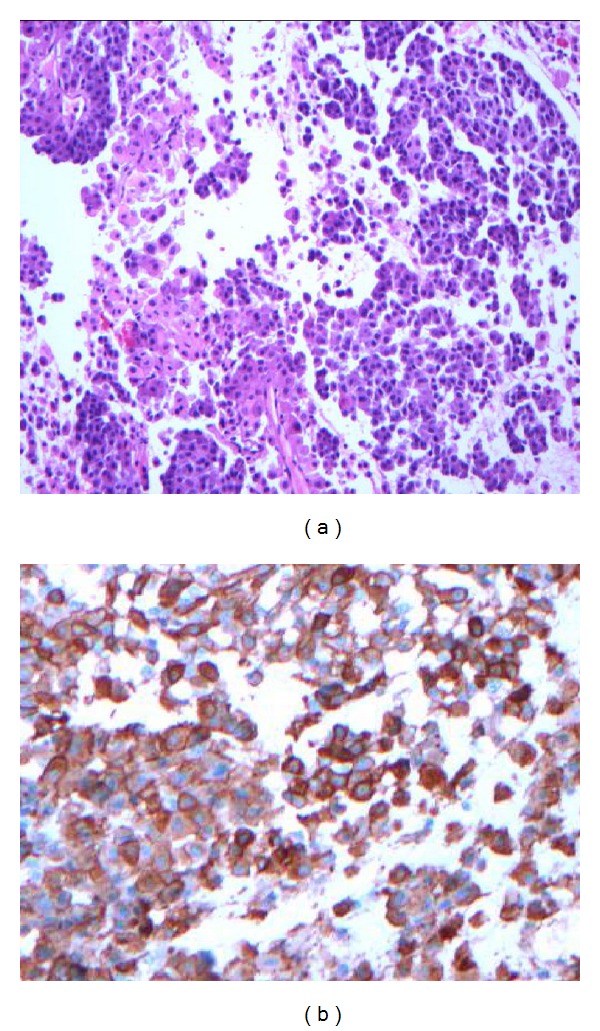
Histology: (a) H&E stain showing basophilic cells at magnification ×200. (b) Positive ACTH staining.

A 38-year-old female noticed progressively worsening bilateral proptosis for a period of two years, to the point causing episodes of ocular dislocation from her sockets. She also noted irregular menstrual cycles during this time and was amenorrheic for 6 months prior to referral. She underwent extensive workup by her primary care physician including thyroid tests which were normal. She then underwent orbital decompression surgery in June 2011 with transient improvement of symptoms. However in the next 12 months she gained 60 lbs and developed proximal muscle weakness, purplish abdominal striae, facial hirsutism, and easy bruisability. She was also diagnosed with new onset diabetes and hypertension during this time and was treated with Metformin and Lisinopril, respectively. Physical examination revealed an obese female with a BMI of 43 and BP 126/78. She had frank stigmata of Cushings syndrome (CS). She had bilateral proptosis with Hertel's exophthalmometry readings of 26 mm (right) and 27 mm (left) (Figure 1). Visual acuity was 20/60 bilaterally. There was no corneal/conjunctival congestion or lid retraction/lag. Fundus exam was normal. Extraocular movements were intact and visual fields were normal on confrontation. Tonometry was not performed. Labs done prior to referral indicated midnight salivary cortisol of 654 ng/dL (normal <112 ng/dL) and post 1 mg dexamethasone cortisol of 16.9 mcg/dL. Random ACTH level was 50 (8–42 pg/mL). MRI of pituitary gland revealed 1.6 cm macroadenoma with deviation of the stalk to the right (Figure 2). MRI also indicated bilateral exophthalmos with increased retrorbital fat (Figure 2). Prolactin was 40 (2–17.4 ng/mL) consistent with stalk effect, gonadotropins were low, and IGF-1, free T4 were normal. Patient underwent trans-sphenoidal removal of the tumor which stained diffusely with ACTH (Figure 3). Patient is being treated with hydrocortisone and followed closely by her ophthalmologist. Although the exophthalmos persisted after the pituitary surgery, episodes of ocular dislocation had not occurred at 3 months followup.

## 2. Discussion

Exophthalmos or proptosis refers to forward displacement of the eyeball. It has to be differentiated from retraction of the eyelids, which can cause an illusion of exophthalmos. Conventionally, exophthalmos refers to ocular proptosis secondary to endocrinopathies. Graves' disease is the most common endocrine cause of exophthalmos. Although described in 1932 by Harvey Cushing in 4 of his 12 patients with Cushings disease, this is an often forgotten clinical sign [1] in patients with CS. We have presented a case highlighting the importance of exophthalmos and its association with hypercortisolemia.

Exophthalmos is seen in about 30–45% of patients with Cushings syndrome (CS) [1–3]. Kelly reported that exophthalmos (exceeding 16 mm) occurred in 45% of active CS, 21% of iatrogenic CS, and 20% of treated CS in comparison to 2% in controls [3]. Cases of severe exophthalmos preceding the evolution of CS have been reported in the literature [4, 5]. 

The cause of exophthalmos in CS is still unknown. Multiple theories have been proposed including fat redistribution and increased retro-orbital fat, associated thyroid disease, and an exophthalmos causative factor. It has been proposed that retro-orbital fat deposition is also part of the fat re-distribution seen in CS, resulting in increase in volume of the retro-orbital tissues and a consequent rise in intra-orbital pressure [3, 6]. Orbital fat volume was increased in patients with CS and orbital muscles are relatively spared [7, 8]. In contrast to patients with Graves' disease the retrorbital fat in CS is devoid of inflammatory cell infiltration. Whether differential fat deposition in the orbits is due to increased glucocorticoid receptor density, defective lipolysis or increased lipoprotein lipase activity is not known.
